# Guiding the Implementation of Wastewater-based Surveillance for Carceral Infection Control with Perspectives from People with Lived Experience of Incarceration during the COVID-19 Pandemic

**DOI:** 10.21203/rs.3.rs-4214768/v1

**Published:** 2024-05-08

**Authors:** Victoria M. Brown, Emily A. Ogutu, Alexandra E. Kauffman, Shanika S. Kennedy, Rebecca A. Tenner, Alysse G. Wurcel, Chad J. Zawitz, Anne C. Spaulding, Matthew J. Akiyama

**Affiliations:** Emory University; Emory University; Emory University; Emory University; Tufts Medince; Tufts Medince; Cook County Health; Emory University; Albert Einstein College of Medicine

## Abstract

**Background:**

Little guidance exists on best practices for implementing and sustaining wastewater-based surveillance (WBS) for SARS-CoV-2 in carceral settings. To ensure alignment with priorities of stakeholders, we aimed to understand the perspectives of persons with lived experience (PLE) of jail who were incarcerated during the height of the COVID-19 pandemic on infection control.

**Methods:**

We recruited two PLE at each of four jails: Cook County (IL), Fulton County (GA), Middlesex County (MA), and Washington DC. Focus Group Discussion (FGD) guides followed the Consolidated Framework for Implementation Research (CFIR). Two FGDs focusing on lived experience with jail infection control protocol and WBS were conducted, and six Key Informant (KI) interviews followed to gain insights on communicating WBS results. We used a combination of deductive thematic analysis based on CFIR constructs and inductive analysis to capture emergent themes.

**Results:**

Themes from FGDs included: (1) variable experiences with COVID-19 infection control protocols including intake processes, individual testing, isolation and quarantine, (2) the perceived attitudes of fellow residents and staff surrounding COVID-19 mitigation in a carceral setting; and (3) perceived benefits and challenges involving WBS implementation and messaging. KIs emphasized 1) The importance of straightforward health messaging and trustworthiness in the communication of WBS results, 2) Support for enhanced health education around outbreaks, and 3) Receptiveness to WBS being used as a tool to measure common infectious agents (i.e., influenza) but hesitancy regarding its application to conditions such as HIV and illicit drug use. PLE articulated support of robust infection control programs and receptiveness to expanding WBS if conducted in a non-stigmatizing manner.

**Conclusion:**

Perspectives from PLE can help shape the infection control programs for future outbreaks and inform the expansion of WBS implementation in carceral facilities. It will be important to consider the voices of current and former residents, as receivers of care, to promote an environment conducive to comprehensive infection control. In addition to having infection control programs consistently execute set protocols and educate all stakeholders, PLE identified collaboration between jail staff and residents, and clear communication around program expectations as priorities. Findings from this qualitative study can be shared with jail decision makers and the perceived engagement of stakeholders can be measured.

## Background

Beginning in 2020, the COVID-19 pandemic ferociously hit U.S. jails, which are institutions for those awaiting trial or serving short sentences.^1^ Mitigating COVID-19 outbreaks in these facilities was a challenge.^2^ Members of this writing group have previously published results of the correlation of Wastewater-based surveillance (WBS) results and the proportion of individuals incarcerated in a jail infected with SARS-CoV-2, the virus that causes COVID-19.^3^ We have further demonstrated that WBS can provide granular information on specific areas in the jail where outbreaks occur and thus save time, labor, and resources in mitigation efforts.^4^ Jail facilities face barriers to infection control such as overcrowding, low resources, and competing demands of safety and health. Innovative surveillance tools to help manage infectious diseases can address the barriers. Nonetheless, sustaining WBS in jail infection control protocols requires rigorous study to outline effective implementation strategies and the support of diverse stakeholders.

To investigate the complexities of WBS implementation and sustainability in jails, Emory University, in a cooperative partnership with the National Institute of Health and community partners in its RADx program for underserved populations (RADx-UP) launched a study— Conducting Correctional COVID Research and Implementing Novel, Ethically Sound, Sustainable Surveillance Systems (CRAINES, U01-DA056000) in 2022. The CRAINES study created implementation teams and sought representation from leadership, middle management, and line custodial and medical staff. CRAINES also included persons with lived experience (PLE) of incarceration during the COVID-19 pandemic at each of four jails: Fulton County (Atlanta, GA), Cook County (Chicago, IL), Middlesex County (Billerica, MA), and Washington DC.^4^ The decision to include PLE as members of the implementation teams is supported by principles of community engagement and collaboration. Carceral health administrators have historically overlooked the voices of the incarcerated. The focus of this paper is to understand the perspectives of PLE regarding infection control and WBS in the context of COVID-19. Its aim is to demonstrate whether engaging diverse stakeholders, including those who are typically not included, could yield valuable insight and feasible suggestions in improving infection control and the implementation of WBS in a carceral setting.

## Methods

The study appointed site leads for each of the four jails. In the fall of 2022, an appointed site lead at each of the jails recruited two PLE who had resided at their respective jail sites at some time during the COVID-19 pandemic. PLE were recruited predominantly from jail transition clinics or advocacy groups. In a general orientation webinar for these participants, we provided education on WBS and infection control strategies. We explained that the study was seeking input from people with lived jail experience on how an infection control strategy, incorporating WBS, could be used in the future. During this meeting, participants independently completed an interactive WBS worksheet (Supplement 1), then engaged in group dialog to discuss their answers to further familiarize participants with WBS and the stakeholders surrounding its implementation in carceral settings.

We conducted two focus group discussions (FGDs) for PLE on Zoom with enrolled subjects to explore their experiences with COVID-19 mitigation, suggestions for implementation of WBS, and improvement in infection control protocols. The FGD guides were developed using the Consolidated Framework for Implementation Research (CFIR) framework.^5,6^ The eight participants were permitted to participate in one or both groups at their choice.

The FGDs were facilitated by a trained moderator with diverse experience in conducting qualitative research. The moderator guided the discussions, and a trained notetaker took notes and recorded the discussions. Each FGD began with a reintroduction of the scientific context of WBS. Participants shared their experiences in jail during COVID, and made suggestions for COVID-19 mitigation, implementation of WBS, and improvement in infection control protocols. The audio recordings were professionally transcribed, and transcriptions were proofread for accuracy by the research team. The audio recordings were deleted upon completion of transcription.

After the FGD, we conducted individual key informant (KI) interviews with participants to gain further insight into best strategies for health messaging for WBS, improving protocol adherence, and expanding WBS to other pathogens. Questions focused on how WBS results could be presented to detained populations. We shared two types of figures with each participant: a redacted heat map of outbreak sites within a jail, and a lower literacy notification of wastewater levels of virus. (Fig. 2). KI interviews were facilitated by 1–2 trained member(s) of the study team. Data collection and transcription of individual interviews followed the same protocol as the FGDs.

We used Rapid Assessment Processes (RAP) for qualitative analysis.^7–11^ RAP aims to provide more timely results than traditional analysis, to accelerate iterative program improvements and provide information to health care stakeholders over a short timeline. The methodology employs an intensive, team-based, combined inductive/deductive approach using triangulation and iterative qualitative data analysis. Two independent coders from the team of coders (VMB, EAO, SSK, and AEK) reviewed and summarized each transcript using a structured analysis template organized from a *priori* themes to code transcripts. A *priori* themes were created from the interview guide followed during the discussion. Themes that emerged inductively during the analysis were also included. Templates, including extracted quotes, were then iteratively reviewed by the qualitative study lead (MJA) and the rest of the team in periodic study meetings to consolidate and distinguish themes. In these meetings, we ensured alignment and resolved discrepancies. Final templates were used to generate and refine key learnings and identify themes with core qualitative investigators (VMB, EAO, SSK, AEK, and MJA).

The CRAINES study was approved by the Emory Internal Review Board. All participants provided written consent before participating in the overall study and provided a verbal affirmation before the start of the FGDs and individual interviews. Compensation was provided for participant time.

## Results

**Figure 1.**
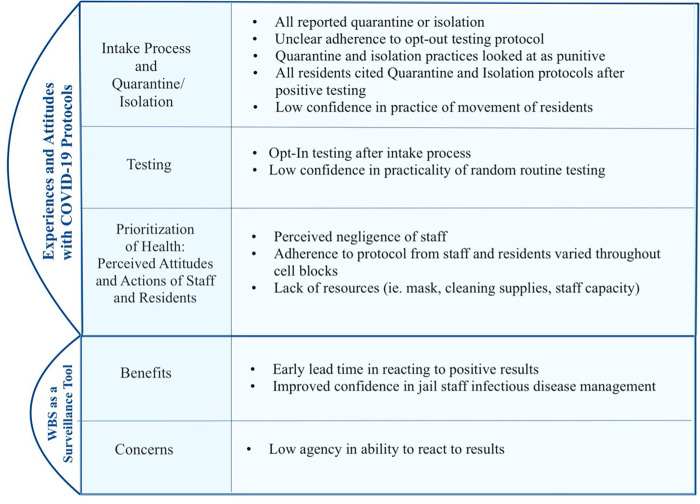
Summary of Focus Group Discussion with Formerly Incarcerated Participants

All eight participants attended at least one focus group. Four to six PLE in the four jails of interest participated in each of the FGDs. Six participated in KI Interviews; two participants did not respond to our study team at the time of scheduling interviews and did not participate in the interviews. Key themes from the FGD included: (1) variable experiences with COVID-19 infection control protocols including intake processes, individual testing, isolation and quarantine, (2) the perceived attitudes of fellow residents and staff surrounding COVID-19 mitigation in a carceral setting; and (3) perceived benefits and challenges involving WBS implementation and messaging. In the first section and Fig. 1, we outline these overarching findings. In section two, findings were summarized from the KI Interviews on (1) health messaging, (2) education, and (3) expansion of WBS to other detectable pathogens and illicit drug use. In this report we will focus on the qualitative data about infection control, rather than general conditions of the jail during COVID-19.

Experiences and Attitudes with COVID-19 Protocols in a Carceral Setting

### 1. Intake Process and Quarantine/Isolation Protocols–Leading to Distrust

In the FGDs, participants began by discussing their experiences with COVID-19 infection control protocols while incarcerated. Participants reported that the jails they resided in screened newly incarcerated individuals for COVID-19 before integrating them into their general population. Each of the four jails had an official intake protocol which included testing and a period of either isolation or quarantine in the event of a positive test. In speaking about testing, some participants highlighted inconsistencies in COVID testing as part of their intake process.

“They’re supposed to do [testing] downstairs in intake, but I done had guys come in my cell, and I’d be like, boy, did you get tested before you came to the cell? And he’d be like, nah, they ain’t do no test on me.”

—Participant #1

Participants had low confidence in how jail staff adhered to their own isolation and quarantine protocol. Staff appeared to enforce protocols inconsistently. Discussants expressed their belief that the staff mismanaged the movement of residents. They indicated their perception that jail staff lost track of where residents were located within the facility and did not have clear methods of moving residents in and out of isolation/quarantine.

“[If] they said one of the inmates had COVID, all they would do is remove the inmate from the unit and send him wherever they send them at and lock the rest of the unit down. But they didn’t like come cell to cell worrying about who else might have COVID or none of that.”

—Participant #1

Participants recognized the constant influx and relocation of residents as a potential barrier of protocol execution. Several believed their facilities were consistently overcrowded, which may have hindered proper isolation and quarantine practice.

“[I] just think they can’t even quarantine people right in jail, because it’s just too crowded.”

—Participant #4

Former residents discussed their frustration with quarantine and isolation protocols due to their similarities to punitive protocols such as lockdown. Both medically induced isolation/quarantine and custodial induced lockdown protocols minimize time outside of the cell. In discussing quarantine, participants used the term lockdown interchangeably with quarantine to describe their state of movement and freedom within their cell block. Participants expressed that being on lockdown, especially so often, led to low social interaction and physical movement, which they believed caused negative impacts on their mental health.

“I had a lot of anxiety attacks, and I’ve been locked up all my life, but being punished with something that’s nothing I did is messing with your psyche.”

—Participant #8

### 2. Issues with Ongoing Individual Testing for SARS-CoV-2

According to participants from all facilities, only positive test results were communicated with the residents. Some reported having preferred knowing their test results, even if it were negative, to quell testing anxiety. The usefulness of mass testing was questioned in the discussion, citing that mass testing within larger facilities still only selected a portion of the population at a time due to lack of capacity of resources. Incremental mass testing strategies left residents less confident in their individual test results given how quickly infection can spread.

“…Testing was done bit by bit, so today you test somebody, you find the results to be negative, and you cannot know tomorrow.”

—Participant #3

“Yeah, pretty much after intake it was a wrap. If you wanted another test, you’ve got to request it.”

—Participant #4

### 3. Prioritization of Health: Perceived Attitudes and Actions of Jail Staff and Residents

Throughout the FGDs, participants discussed themes surrounding their perception of the prioritization of health from fellow residents and jail staff. Many expressed frustrations with jail staff for perceived negligence towards residents and their health. When discussing jail staff, participants focused on staff in custodial positions, as they are the primary staff who engage with residents, as opposed to medical staff.

As an example of the perceived neglect, participants discussed shared experiences of limited cleaning efforts and lack of materials such as bleach and disinfectant. Some shared that there were not enough masks made readily available. They also detailed a slow process to connect with health services.

“They put us at risk as far as health-wise and everything. Like they just didn’t care. That’s all I can really say. They just —they didn’t care.”

—Participant #8

“You would think they would be passing out cleaning supplies, you know, being so, you know, COVID is going on.”

—Participant #2

Many reported they had to advocate for themselves to any jail staff they encountered for additional testing or medical services. In discussing this, participants acknowledged that jails often face issues of understaffing and limited resources. Some were empathetic with the jail staff given the lack of capacity and resources in such dire times and offered support to improving conditions.

“The jails are so understaffed, so when they have something extra to do, it ain’t going to get done or not get done properly.”

—Participant #4

“I can assist any who is there, even [the] medical director I can give useful information…because I have the experience of being at the jail.”

—Participant #3

Regarding attitudes of fellow residents, participants mentioned topics of testing and quarantine fatigue. Participants reported that attitudes towards continuous quarantine were so negative that there were negative reactions towards people who were exhibiting symptoms of COVID-19 within a cell block, given the implications that their symptoms would trigger the lockdown protocols. Moreover, misinformation about COVID-19 during the pandemic may have also resulted in lower adherence to recommended CDC COVID-19 mitigation behaviors, such as masking, from some residents.

“Some people [residents] took it serious and some didn’t. I took it serious from jump. You know, I don’t play no games with no – dealing with no diseases. At all.”

—Participant #8

#### Attitudes and Beliefs on Wastewater as Surveillance as a Tool

Participants expressed interest in WBS as a strategy for COVID-19 surveillance in their jail facility. Discussants recognized the benefits of including WBS into jail systems such as early detection, enhanced testing strategies, and better allocation of resources. The former residents expressed their belief that WBS results should be made available to residents—citing the early lead time in detecting virus as useful. Participant #5 voiced that with knowledge of community viral levels, residents “could plan ways to protect themselves from acquiring [the] COVID-19.” Others shared their concerns that sharing WBS results could result in fear among the general population. Those who voiced this concern explained that WBS results would not be beneficial to them since they have no agency in how they could respond to those results; it would be up to jail staff to respond to an outbreak.

“If something new can be pushed out there and help scientists or whatever, you know, again, I think it’s worth a shot. Anything that has to do with our health and moving forward I think is always interesting.”

—Participant #2

“That shouldn’t be an inmate’s responsibility, because basically an inmate can’t do anything about it.

—Participant #4

#### Health Messaging

**Figure 2:**
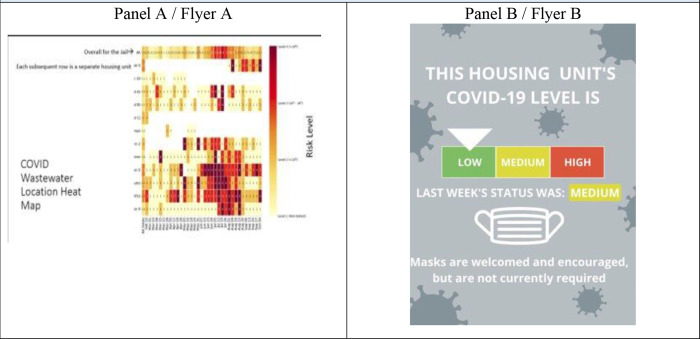
Communication Styles of WBS Results Formerly Incarcerated Participants were Probed on, Individual Interviews. (A) a redacted heat map that had been utilized by Cook County Health among jail staff and the infectious disease team. The study team facilitator highlighted the titles of the axes of the heat map, providing an orientation to the figure before soliciting feedback; and (B) a flyer coding housing unit levels by color, based on its corresponding wastewater level, similar to CDC COVID-19 Community Levels that were introduced in February 2022. (CDC Media Telebriefing: Update on COVID-19 | CDC Online Newsroom | CDC) An arrow pointed to where on the spectrum risk lay; red levels denoted high risk, yellow medium, and green low risk. Recommendations about masking, a mitigation measure dependent on resident compliance, were included on the flyer.

We solicited insights from subjects on health messaging regarding wastewater surveillance within the carceral setting through show cards. Figure 2 displays two examples of how wastewater results could be shared.

Participants unanimously preferred the flyer in Panel B of Fig. 2, citing the simpler design and colors to be useful in low literacy settings.

“People at the jail, not everybody can read. These people— I can say they like seeing colors and simple communication, like low, medium, and high. Those are common English where they can understand.”

—Participant #3

Though all pointed out the preference of flyer B over flyer A, some participants shared their concerns in using a flyer to communicate WBS results. Participant #1 said “They’ll [residents] tear that paper off the wall and use that to keep score,” when playing cards.

In discussing health messaging, the study team also took the opportunity to ask participants who they believed would be the best communicator of results. Responses varied from outside health officials, custody, floor/cell block nurses, and fellow detained residents. However, it was clear that no matter the suggested entity, trustworthiness was key as expressed by all participants.

“I think they should partner with like the health department or something like that so that the people – so that the inmates know this is legitimate, this is what’s going on, serious, this ain’t no lie.”

—Participant #2

“Results can be even provided by anybody and maybe there are some leaders in jail maybe or a prisoner and they have appointed them to be the leader. You can use those people maybe to communicate. Also, there are caretakers [Custody], yeah, you can use caretakers to communicate...you will find these people most of the time they are with the prisoners.”

—Participant #3

#### Education

All participants recognized the potential benefits of increasing education about COVID-19 including symptoms, mitigation, and protocol.

“...the most important thing that [can] help us was education, awareness about the COVID-19. After they educate us on various measures that we can do, many people take heed of that”

—Participant #3

#### Expanding WBS to other Detectable Pathogens and Illicit Drug-use

In considering the longevity of a wastewater surveillance program, we asked participants about their thoughts on expanding WBS programs to additional pathogens. In theory, testing for additional pathogens would be a relatively small laboratory ask. However, we found that surveilling for additional pathogens may require more thoughtful implementation.

We asked each participant about expanding surveillance of wastewater to the influenza virus, HIV, and illicit drug use. Overall, the reactions to testing for the influenza virus mirrored the those of testing for SARS-CoV-2; the jail could benefit from the additional information WBS can provide. The feedback on HIV and illicit drug use surveillance was mixed. Though former residents agreed with the importance of infection control for HIV, many commented on the stigma that is associated with HIV.

“You can’t let a group know that somebody’s dirty, you know what I’m saying? Somebody’s positive. Because the group of inmates might not take it as well.”

—Participant #4

All participants were reluctant to fully support WBS for HIV. Privacy was continuously mentioned, with participant #3 adding “testing each individual and giving them results... keep[s] the privacy of these prisoners” when comparing utilizing WBS for HIV surveillance to traditional individual testing. Several participants also questioned the ethics behind communal testing for HIV.

In asking about WBS for illicit drug-use, several participants had follow-up questions on how results would be interpreted, given the high churn of jails and staff also having access to restroom facilities.

“I might need example [of interpreting time in the context of surveilling drugs]. I can take these drugs, maybe at the street, tomorrow maybe I’ll be jail – I’ll be jailed, and when you test me at the jail maybe tomorrow you can find I am positive and you can suspect some people are using or some prisoners are using these drugs, but that is not true.”

—Participant #3

## Discussion

Persons jailed during the COVID-19 pandemic offered valuable insights and innovative ideas in mitigating outbreaks. The input of persons who experienced confinement makes us recognize that adherence to principles of consistency, clarity, and collaboration is imperative to improve infection control. Though they recognized the value of protocols such as isolation, quarantine, and testing, residents expressed low confidence in COVID-19 control within their respective jails.

The FGDs highlighted perceived inconsistencies in testing and quarantine policies. Participants reported some perceived breaches in protocol came from staff not supporting what measures could be enacted for SARS-CoV-2 mitigation. Other perceived deviations arose due to shortages in PPE. Participants also described perceived inconsistent and disorganized movement of individuals. None of the former residents expressed confidence in their jail’s infrastructure in handling confirmed cases and exposed individuals. The complexity of the relationship between residents and carceral heath systems is being newly developed in the literature.^12^ Studies have found individuals who are incarcerated often report sub-standard care and PLE score high on the Revised Healthcare System Distrust scale, indicating they may have decreased distrust in health systems in general.^13^

Though participants recognized the use of isolation and quarantine in reducing transmission, they also communicated frustration with existing protocols, given their already limited mobility and agency. Quarantine and isolation protocols mimicked punitive protocols (i.e., lockdown) so closely that participants used the words interchangeably. Such blurred relationship between residents with medical and custodial protocols may worsen attitudes towards infection control. The relationship between incarcerated persons and social distancing by way of lockdown is recognized as punitive in the literature, and implementation of social distancing throughout lockdown must further be considered by public health institutions and local, state and federal authorities.^14^ Since U.S jails rely on tight group housing, single-cell housing is often the only way custody can attempt social distancing. The negative attitudes surrounding lockdown as quarantine and isolation protocols, shared by former residents, may negatively affect adherence and symptom reporting in real-time. Rewarding quarantined residents with additional recreation, products from the commissary, and/or entertainment could diminish the punitive feel of isolation. Promptly cohorting people who test positive may also diminish the association between medical isolation and punishment. Sequestering the infected from those susceptible should not insinuate that the biological state of infection, rather than misbehavior, merits punishment.

While participants shared their criticism, many did empathize with the jail staff on the difficulty of harmonizing infection control and custodial protocols in under-resourced jails. Their thoughtful consideration of jail management signifies a reconciliation in the relationship between residents and jail staff. Participants demonstrated they want to contribute to a change in infection control policy—they wish to collaborate on implementing innovation.

Participants had thoughtful insight on how infection control could be innovated within jail systems. They shared ideas on promoting health education, health messaging, and WBS. Former residents had strong preferences about how to communicate COVID risk, who are trusted messengers of health-related information, and what viral diseases should be monitored by WBS. They were able to discuss the potential benefits and challenges of expanding WBS to additional viral pathogens. Although they saw the benefit in WBS as an additional surveillance method beyond mass testing, participants also brought up privacy and stigma concerns, which could be further heightened in a jail setting.

### Call to Action: Consistency, Collaboration, and Clarity of Communication

In considering the areas for improvement discussed by PLE in our study, we recommend jail systems strengthen their infection control programs by consistently executing set protocols and rapidly adapting said protocols when scientific understanding changes and expert recommendations prompt amendment. Jail systems should support collaboration between medical staff, custodial staff, and residents, and provide clear communication in forms of infection control education and expectations.

Jail health systems and custodial leaders will need to collaborate with each other to increase the coordination of the rapid movement and relocation of residents within the facility. Results found custodial officers are major players in COVID protocol adherence within a cell block. Residents were mindful of the low resources in spacing and staffing available in a jail setting and may be more amenable to consistent and clear relocation efforts. Clear and consistent communication and action between infection control roll-out and practice could improve trust in the carceral health system.

Additionally, the healthcare team working in corrections should recognize residents support infection control education that they can trust. Misinformation and lack of health information in a pandemic hinders infection control. Residents have low agency to pursue their own disease education. Trusted messengers communicating clearly about current risk level builds a sense of collaboration in mitigating disease. These could include signage, announcements, and active teaching by various trusted communicators on disease management and infection control.

Furthermore, jails should also consider expanding WBS health messaging that reaches residents. Knowledge that the wastewater signals are high versus low can spur enhanced mitigation action. Maintaining distances from others, vigilance in covering coughs, and wearing masks all depend on individual agency. When communal viral levels are high a resident can respond by intensifying their mitigation practices and when viral levels are low, they can relax their behavior. Having this information accessible could lessen pandemic fatigue and improve trust of jail staff’s enforcement of infection control protocol. Many informants believed that the results of WBS should not remain solely in the hands of the healthcare staff.

Former residents were able to distinguish the difference between how the infection control program was ideally supposed to function, and its actual roll out. Carceral leadership has historically overlooked voices of PLE when developing communicable disease protocols. However, as receivers of care, PLE’s input into these programs is critical to improving infection control management for future outbreaks. PLE’s insights are important to successful implementation and expansion of infection control protocols in carceral settings. Participant #3 offer of help to the medical director (see section 4) encapsulates the importance of jail residents’ potential contributions to future innovations of enhanced infection control programs. All stakeholders should have a role in implementing innovation.^15,16^ New implementation science tools that help promote broad community engagement are ones that measure the receptivity of policy makers to input from these forgotten partners, PLE. Findings from this qualitative study can be shared with jail decision makers. Our final step will be measuring the perceived engagement of all stakeholders.^17–19^

## Definitions

Cell block: a housing unit comprised of multiple cells for multiple persons, usually with a common area.
Lock down: a state of movement restrictions facilitated by custodial staff that can enhance security. This is often enacted after behavioral violation(s) or breaches of security.
Residents: persons detained and residing overnight in a jail. We refrain from terms like detainees and offenders, which refers to persons by their legal status.
